# Heart rate, blood pressure, and SpO_2_ responses to simulated 5000 m hypobaric exposure in healthy male Vietnamese pilots

**DOI:** 10.14814/phy2.70733

**Published:** 2026-02-11

**Authors:** Xuan Nguyen Thanh, Phong Nguyen Hong, Tuan Tran Ngoc, Phuong Nguyen Minh, Toan Pham Quoc, Toan Nguyen Duy, Thao Pham Ngoc, Thong Nguyen Huy, Luyen Nguyen Van, Thuc Luong Cong

**Affiliations:** ^1^ 103 Military Hospital, Vietnam Military Medical University Hanoi Vietnam; ^2^ Vietnam Military Medical University Hanoi Vietnam

**Keywords:** altitude physiology, autonomic regulation, aviation medicine, cardiovascular response, hypobaric hypoxia, oxygen saturation

## Abstract

This study was motivated by the limited high‐altitude physiology data available for Asian military aviators, especially in Vietnam. To characterize acute heart rate (HR), blood pressure (BP), and oxygen saturation (SpO_2_) responses in healthy Vietnamese pilots during simulated 5000 m hypobaric exposure. Seventy‐five healthy male military pilots underwent 30‐min exposure in a hypobaric chamber simulating 5000 m altitude. HR, systolic/diastolic BP (SBP/DBP), and SpO_2_ were recorded at baseline (0 m), peak altitude, and post‐exposure. HR increased from 80.0 ± 9.0 to 91.9 ± 11.0 bpm, SBP/DBP rose by 10.4/6.5 mmHg, and SpO_2_ decreased from 98.4 ± 1.0% to 77.7 ± 6.0% (all *p* < 0.001). All changes were transient, returning to baseline within 5 min after exposure, and no adverse events occurred. Higher baseline HR and older age predicted smaller HR increases. Acute 5000 m hypobaric hypoxia caused transient tachycardia, hypertension, and hypoxemia. Response variability correlated with baseline hemodynamic status and age. This safe, reproducible model can serve as a noninvasive cardiovascular stress test in clinical and aerospace medicine.

## 1 INTRODUCTION

Exposure to high altitude (low barometric pressure) elicits well‐characterized physiological responses. As ambient oxygen partial pressure falls with increasing altitude, peripheral chemoreceptors stimulate hyperventilation to improve oxygen uptake (Taylor, 2011). Acute hypoxia triggers sympathetic activation and vagal withdrawal, leading to tachycardia (increased heart rate), enhanced myocardial contractility, and elevated cardiac output. Blood pressure responses can vary: many individuals exhibit an initial rise in arterial pressure due to increased cardiac output and systemic vasoconstriction, although some studies note that systemic vascular resistance may decrease at altitude, which can moderate the overall blood pressure response (Theunissen et al., 2022). Consequently, arterial oxygen saturation (SpO_2_) declines as altitude increases. In unacclimatized subjects at approximately 3800 m, SpO_2_ typically falls to the mid‐80% range (Theunissen et al., 2022), and at 5000–5500 m, it can drop into the 70%–80% range. At extreme altitudes near 7000 m (≈22,000 ft), SpO_2_ may reach around 60% or lower (Alvear‐Catalán et al., 2024), which can precipitate significant neurocognitive impairment if supplemental oxygen is not provided. Despite extensive high‐altitude research in Western and other populations, data on Asian subjects—particularly pilots—remain limited. Most published studies of hypoxia training and physiology have involved North American or European cohorts (Alvear‐Catalán et al., 2024). In Asia, some studies have examined hypoxia effects during altitude chamber tests in pilots (Kasture et al., 2021), but comprehensive physiological response data from Southeast Asian aircrew remain scarce. To our knowledge, no prior study has specifically quantified cardiovascular and respiratory responses to altitude in Vietnamese aircrew. This represents a critical gap, as ethnic and regional differences (in baseline health, genetics, or environment) could modulate altitude tolerance. This study aimed to characterize the acute cardiovascular and oxygenation responses to simulated high‐altitude hypoxia in a Vietnamese pilot cohort. Healthy military pilots were exposed to a hypobaric chamber profile simulating 5000 m (≈16,400 ft)—an altitude at which hypoxia symptoms can occur but which is still safely within training limits (Alvear‐Catalán et al., 2024). Heart rate, blood pressure, and SpO_2_ were measured at baseline, during altitude exposure, and after descent. By comparing subgroups (BMI ≥23 vs. <23 kg/m^2^, and smokers vs. nonsmokers) and examining correlates such as age, we sought to identify any modulators of individual susceptibility. These findings provide novel data on an Asian pilot population that can inform aeromedical training and risk stratification for high‐altitude operations.

## 2 METHODS

### 2.1 Study design and participants

This cross‐sectional observational study was conducted at the Vietnam Institute of Aviation Medicine in 2024. Seventy‐five healthy male pilots (mean age 37.9 ± 8.5 years; BMI 24.5 ± 2.2 kg/m^2^) were recruited during routine altitude chamber training. All were on active flying status with no known cardiopulmonary or hematologic diseases. Based on WHO Asian BMI criteria, 84% were overweight (BMI ≥23), and 40% were current smokers. Participants underwent comprehensive medical screening including clinical examination, routine blood and urine tests, chest X‐ray, and vestibular testing to ensure eligibility for altitude exposure. All participants provided written informed consent. The study protocol was approved by the Institutional Review Board (IRB) of the Vietnam Military Medical University (Approval No. 4050/QĐ‐HVQY, dated 20/09/2024). The study was conducted in accordance with the principles of the Declaration of Helsinki.

### 2.2 Altitude exposure protocol

Altitude exposure was performed using the HPO 6 + 2 hypobaric chamber system (AMST, Austria). Prior to exposure, participants were re‐screened for resting blood pressure, body temperature, and pulse, and briefed on safety procedures.

The protocol included four main phases: (1) Preparation: Subjects were seated in assigned chamber positions and connected to a physiological monitoring system (Philips IntelliVue MX750). Safety checks and communication systems were verified; (2) Ascent: The chamber ascended from ground level to 5000 m at a rate of 10–15 m/s over approximately 5–6 min; (3) Exposure: At 5000 m (PB ≈ 405 mmHg, FiO_2_ ≈ 11.3%), pilots remained for 30 min breathing ambient air without supplemental oxygen. SpO_2_, HR, and symptoms were closely monitored every 15 min and whenever adverse signs appeared; *(4)* Descent: The chamber returned to baseline over ~5 min. Afterward, pilots were monitored for at least 10 min to ensure recovery and absence of residual effects.

No early terminations occurred, and the procedure was overseen by trained aerospace physicians and technicians.

### 2.3 Physiological measurements

Key variables—heart rate (HR), oxygen saturation (SpO_2_), systolic (SBP), and diastolic blood pressure (DBP)—were recorded at three main time points: Baseline (0 m, before ascent); Peak exposure (5000 m); Post‐exposure (0 m, after descent).

HR and SpO_2_ were continuously monitored via fingertip pulse oximetry. The minimum SpO_2_ and corresponding peak HR values at 5000 m were selected for analysis. SBP and DBP were measured using an automated sphygmomanometer at baseline, early altitude (1–2 min), and at ~20 min during exposure; the highest altitude value was used. Post‐exposure vitals were recorded within 1–3 min after descent.

Additional readings of HR and SpO_2_ were recorded incrementally during ascent at 1000, 2000, 3000, and 4000 m, and at 0, 15, and 30 min during the 5000‐m exposure.

Anthropometric data were collected to calculate BMI. Smoking status was self‐reported. Flight experience data (e.g., total flight hours) were documented but not analyzed in this study.

### 2.4 Statistical analysis

Data were analyzed using SPSS (v26) and R (v4.3.2). Continuous variables are presented as mean ± SD, and categorical variables as counts or percentages. Changes in heart rate (HR), blood pressure (BP), and oxygen saturation (SpO_2_) across time points (pre‐exposure, altitude, post‐exposure) were evaluated using repeated‐measures ANOVA with Bonferroni correction for post hoc comparisons.

To enhance robustness, we also conducted sensitivity analyses using linear mixed‐effects models (LMMs), treating subject ID as a random effect and time and smoking status as fixed effects. These models confirmed the direction and significance of the ANOVA findings.

Between‐group comparisons (e.g., BMI ≥23 vs. <23; smokers vs. non‐smokers) were performed using independent *t*‐tests. Pearson correlation assessed associations among baseline and response variables. Multivariate linear regression identified independent predictors of HR increase and SpO_2_ decrease. Covariates included age, BMI, and smoking status. Assumptions of linearity, normality, and multicollinearity were checked and met.

A two‐tailed *p*‐value <0.05 was considered statistically significant. The sample size (*n* = 75) provided >90% power to detect a ≥10 bpm change in HR or ≥5% change in SpO_2_.

## 3 RESULTS

### 3.1 Baseline characteristics

The study population consisted of 75 healthy Vietnamese male pilots (mean age: 38.0 ± 7.5 years), with a mean BMI of 24.8 ± 1.9 kg/m^2^. According to WHO Asian standards, 84% were classified as overweight (BMI ≥23 kg/m^2^). Forty percent of participants were current smokers. Baseline cardiovascular parameters, including SBP (121.3 ± 8.5 mmHg), DBP (75.0 ± 7.1 mmHg), HR (80.0 ± 11.3 bpm), and SpO_2_ (98.4 ± 1.0%), were within physiological norms (Table 1).

**TABLE 1:** Baseline demographic and physiological characteristics of the study participants.

Characteristic	Value
Age (years)	38.0 ± 7.5 (range 24–54)
Height (cm)	169.3 ± 5.8
Weight (kg)	70.2 ± 7.4
Body mass index (BMI, kg/m^2^)	24.8 ± 1.9
Overweight (BMI ≥23 kg/m^2^)	63 (84%)
Current smoker	30 (40%)
Resting SBP (mmHg)	121.3 ± 8.5
Resting DBP (mmHg)	75.0 ± 7.1
Resting heart rate (bpm)	80.0 ± 11.3
Resting SpO_2_ (%)	98.4 ± 1.0

*Note*: Values are mean ± SD or *n* (%).

### 3.2 Acute cardiovascular and oxygenation responses to altitude

**FIGURE 1:**
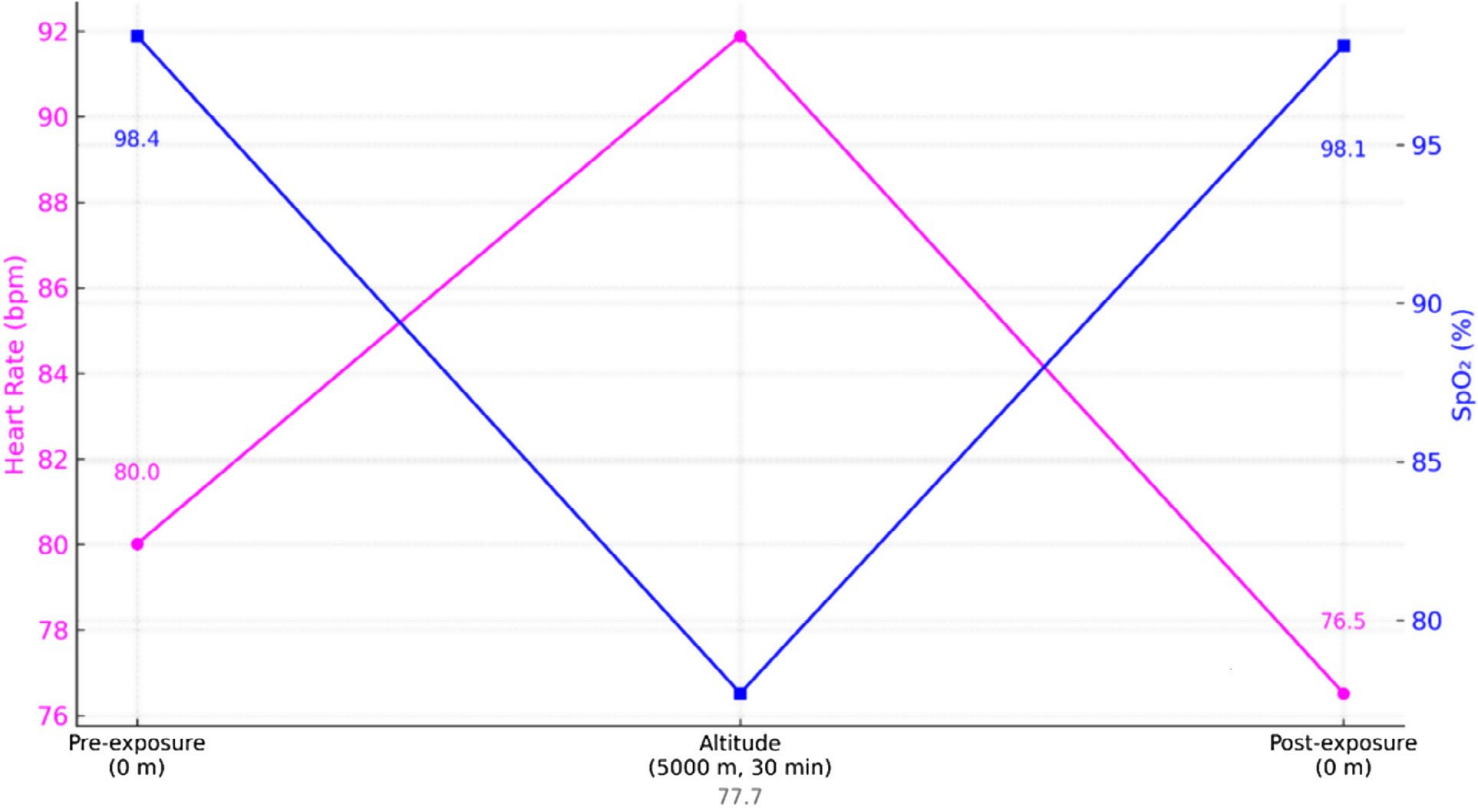
Heart rate and SpO_2_ at baseline (0 m), at 5000 m after 30 min, and post‐exposure (0 m). Magenta circles = heart rate (beats/min); blue squares = oxygen saturation (%). Error bars indicate ±1 SD. High‐altitude hypoxia induced a significant increase in heart rate (*p < 0.001 vs. baseline) and a large decrease in SpO_2_ (*p* < 0.001). Upon return to ground level, values normalized to baseline. Differences in baseline and post‐exposure were not significant.

During hypobaric exposure at 5000 m, participants experienced significant physiological alterations. Heart rate (HR) increased by 11.9 bpm (+14.9%, *p* < 0.001), and SpO_2_ decreased sharply by 20.7% points to 77.7 ± 6.6% (*p* < 0.001). These changes were transient, with full recovery observed within 3 min post descent (Figure 1).

### 3.3 Blood pressure responses at altitude

**FIGURE 2:**
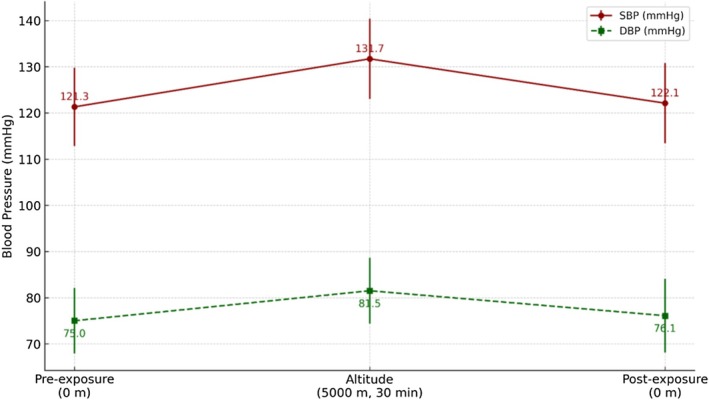
Systolic and diastolic blood pressure at baseline (0 m), at 5000 m after 30 min, and post‐exposure (0 m). Dark red circles = SBP; dark green squares = DBP. Error bars indicate ±1 SD. Both SBP and DBP increased significantly during hypobaric exposure (*p < 0.001 vs. baseline) and returned to baseline levels after descent.

SBP rose from 121.3 ± 12.0 mmHg to 131.7 ± 13.0 mmHg, and DBP increased from 75.0 ± 8.0 mmHg to 81.5 ± 9.0 mmHg during the 30‐min exposure (*p* < 0.001 for both). Pressures normalized after return to ground level (Figure 2).

### 3.4 Overall physiological trends

As summarized in Table 2, all physiological variables—heart rate (HR), systolic blood pressure (SBP), diastolic blood pressure (DBP), and oxygen saturation (SpO_2_)—exhibited statistically significant changes during exposure to 5000‐m simulated altitude (*p* < 0.001 for all) compared to baseline. Following return to ground level, all parameters returned to their pre‐exposure values, with no significant differences observed between baseline and post‐exposure measurements, confirming the transient and reversible nature of these physiological responses.

**TABLE 2:** Heart rate, blood pressure, and oxygen saturation at baseline, altitude (5000 m), and post‐exposure.

Variable	Baseline (0 m)	Altitude (5000 m)	Post‐exposure (0 m)
Heart rate (bpm)	80.0 ± 11.3	91.9 ± 11.0*	76.5 ± 9.0 (n.s. vs. baseline)
SBP (mmHg)	121.3 ± 8.5	131.7 ± 8.7*	122.1 ± 8.7 (n.s.)
DBP (mmHg)	75.0 ± 7.1	81.5 ± 7.1*	76.1 ± 8.0 (n.s.)
SpO_2_ (%)	98.4 ± 0.95	77.7 ± 6.6*	98.1 ± 1.2 (n.s.)

*Note*: Values are mean ± SD. Significance indicated for Altitude versus Baseline and Altitude versus Post‐exposure. No significant differences were found between Baseline and Post‐exposure values for any variable.

Abbreviation: n.s., not significant.

* *p*‐values from repeated‐measures ANOVA with Bonferroni post‐hoc tests. *p* < 0.001.

### 3.5 Associations between baseline characteristics and physiological responses

Table 3 highlights the correlation between individual characteristics and the magnitude of physiological responses. Age and smoking status were inversely correlated with heart rate change (ΔHR), with *r* = −0.25 (*p* < 0.05). Smoking was also significantly correlated with systolic blood pressure change (ΔSBP, *r* = −0.26, *p* < 0.05). No significant correlation was observed between BMI and any variable, nor between any baseline factor and SpO_2_ reduction.

**TABLE 3:** Correlation of pilot characteristics with changes in heart rate, blood pressure, and SpO_2_ during 5000 m exposure.

Predictor	ΔHR (bpm)	ΔSBP (mmHg)	ΔSpO_2_ (%)
Age (years)	−0.252	−0.158	0.036
BMI (kg/m^2^)	−0.172	−0.096	−0.070
Smoker (yes = 1)	−0.255*	−0.263*	−0.048

*Note*: Pearson *r* values.

Abbreviations: ΔHR, HR at altitude – HR at baseline; ΔSBP, SBP at altitude – SBP at baseline; ΔSpO_2_, baseline SpO_2_ – SpO_2_ at altitude.

* *p* < 0.05.

Multivariate regression models identified baseline heart rate as a significant negative predictor of HR increase during hypobaric exposure (*β* = −0.32, *p* < 0.001), consistent with an attenuated response in individuals with higher resting HR. Baseline SBP negatively predicted ΔSBP (*β* = −0.30, *p* = 0.013), and baseline DBP negatively predicted ΔDBP (*β* = −0.39, *p* < 0.001). No baseline factor, including age, BMI, smoking status, or resting values, significantly predicted SpO_2_ decline (Table 4).

**TABLE 4:** Multivariate regression analysis of predictors of physiological response to hypobaric hypoxia.

Predictor	ΔHR (bpm) *β* (95% CI), *p*	ΔSBP (mmHg) *β* (95% CI), *p*	ΔDBP (mmHg) *β* (95% CI), *p*	ΔSpO_2_ (%) *β* (95% CI), *p*
Age (per year)	−0.002 (−0.39 to 0.38), *p* = 0.993	0.14 (−0.14 to 0.42), *p* = 0.327	−0.01 (−0.25 to 0.22), *p* = 0.909	0.20 (−0.11 to 0.51), *p* = 0.201
BMI (kg/m^2^)	−0.73 (−1.75 to 0.29), *p* = 0.160	−0.11 (−0.86 to 0.64), *p* = 0.774	0.25 (−0.37 to 0.87), *p* = 0.417	−0.28 (−1.10 to 0.54), *p* = 0.503
Smoker (yes vs. no)	−2.77 (−8.29 to 2.74), *p* = 0.319	−3.26 (−7.33 to 0.81), *p* = 0.114	0.83 (−2.53 to 4.19), *p* = 0.624	−2.07 (−6.51 to 2.37), *p* = 0.356
SBP baseline	−0.08 (−0.40 to 0.23), *p* = 0.612	−0.30 (−0.53 to −0.06), *p* = 0.013**	0.11 (−0.08 to 0.31), *p* = 0.237	0.01 (−0.24 to 0.27), 0.02, *p* = 0.923
DBP baseline	0.07 (−0.29 to 0.43), *p* = 0.704	0.13 (−0.13 to 0.40), *p* = 0.324	−0.39 (−0.60 to −0.17), *p* = 0.001**	−0.06 (−0.35 to 0.23), *p* = 0.675
HR baseline	**–0.32 (−0.50 to −0.15), *p* < 0.001**	0.00 (−0.12 to 0.13), *p* = 0.951	−0.08 (−0.13 to −0.02), *p* = 0.005**	−0.13 (−0.26 to 0.01), *p* = 0.074

Abbreviations: *β* = unstandardized regression coefficient. CI = 95% confidence interval.

** Statistically significant predictors (*p* < 0.05); Δ = difference from baseline to altitude (5000 m).

**FIGURE 3:**
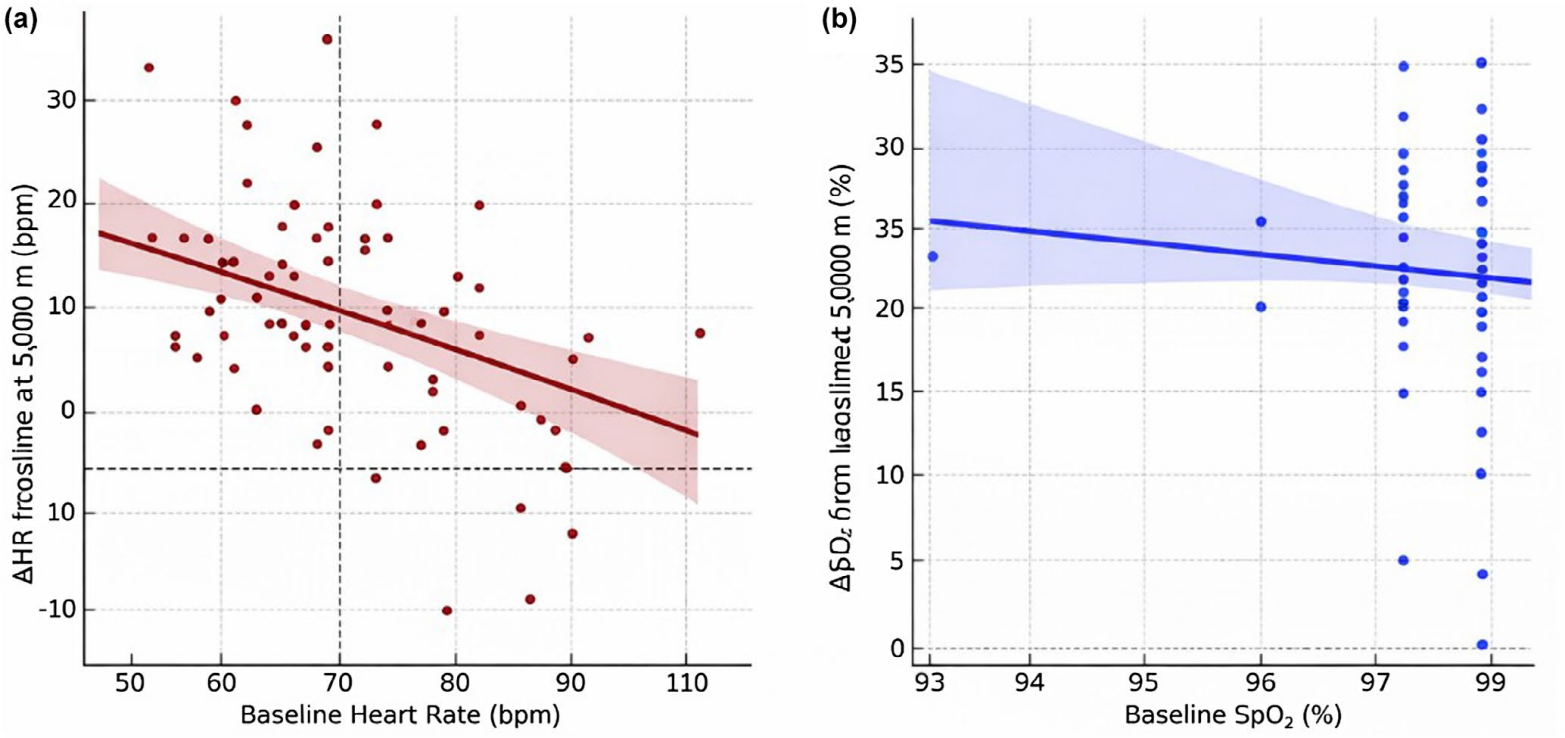
Baseline predictors of physiological response to hypobaric hypoxia. (A) Scatterplot with linear regression showing an inverse relationship between baseline HR and ΔHR (heart rate increase at 5000 m vs. baseline). (B) No significant relationship was observed between baseline SpO_2_ and SpO_2_ reduction. Dashed lines represent mean values; shaded areas indicate 95% confidence intervals. Δ = altitude minus baseline values. Regression models: HR (*R* ^2^ = 0.11, *p* = 0.033); SpO_2_ (*R* ^2^ = 0.03, *p* = 0.57).

Figure 3 illustrates the relationship between baseline physiological values and the magnitude of altitude‐induced changes. A significant inverse linear association was observed between baseline heart rate and the increase in heart rate during exposure to 5000 m (*β* = −0.32, 95% CI –0.50 to −0.15, *p* < 0.001), indicating that individuals with higher resting HR exhibited attenuated responses.

Similarly, baseline SBP and DBP negatively predicted ΔSBP and ΔDBP, respectively. In contrast, no significant baseline predictors were identified for the reduction in SpO_2_ during hypobaric hypoxia. These findings are visually represented in Figure 3a (HR) and Figure 3b (SpO_2_).

## 4 DISCUSSION

This study provides a detailed characterization of acute physiological responses to hypobaric hypoxia (5000 m) in a cohort of healthy Vietnamese pilots. The key findings are as follows: (1) Heart rate increased significantly (~17% on average) with altitude exposure, (2) arterial oxygen saturation dropped dramatically (~21% points) at 5000 m, and (3) blood pressure rose moderately (~8%–9% in SBP/DBP). All changes were transient and returned to baseline shortly after reoxygenation. These results align closely with general principles of high‐altitude physiology and with prior observations in other populations. For example, Fox et al. (2006) reported that acute normobaric hypoxia (12% O_2_, ≈4500 m) induces significant increases in HR and SBP, and we observed similar magnitude increases under hypobaric conditions. Theunissen et al. (2022) found that at ~3842 m, SpO_2_ fell to ~85% while cardiac output rose ~43% (driven largely by a ~17% HR increase); in our study, the 5000‐m profile produced a larger SpO_2_ decline (~77% mean nadir) with a comparable HR increase (~17%), consistent with the greater altitude stress. Similarly, Kasture et al. (2021) reported increased HR and decreased SpO_2_ at 22,000 ft. (6700 m), qualitatively paralleling our findings (although at 6700 m SpO_2_ is typically <70% and symptoms are more severe). Taken together, these results reinforce that the cardiopulmonary system reacts robustly to acute hypoxia, with tachycardia, modest hypertension, and pronounced O_2_ desaturation as hallmark compensatory responses. An interesting contrast lies in blood pressure. Some prior studies have reported minimal changes or slight decreases in systemic blood pressure during acute hypoxia in resting subjects (Theunissen et al., 2022), attributing this to hypoxia‐induced vasodilation (reduced systemic vascular resistance). In our cohort, however, both SBP and DBP increased significantly at 5000 m. This suggests that sympathetic activation (and possibly mild anxiety or exertion during chamber exposure) outweighed any vasodilatory effects, resulting in a net pressor response. Methodological factors likely contribute: for example, Theunissen et al. (2022) observed a drop in mean arterial pressure at 3800 m when subjects were supine with gradual decompression, whereas our pilots were seated during a faster ascent—a scenario that can provoke a stronger adrenergic (orthostatic) response. Notably, some individuals in our sample showed little BP change, indicating variability. Overall, these data support the notion that acute hypobaric hypoxia tends to modestly elevate blood pressure, in agreement with Fox et al. (2006) and studies of intermittent hypoxia (Wszedybyl‐Winklewska et al., 2017) that report pressor effects via chemoreceptor‐mediated sympathetic activation.

We did not observe significant differences in hypoxic responses based on BMI. This finding is consistent with the narrow BMI range in our cohort (mean BMI: 24.5 ± 2.2 kg/m^2^; mostly between 22 and 27 kg/m^2^), and the absence of obese individuals. While obesity can impair respiratory function and reduce ventilatory reserve, thereby worsening hypoxemia at altitude (Martin et al., 2017), our sample likely consisted of moderately overweight yet physically fit pilots with relatively preserved cardiorespiratory function (e.g., greater lean mass and aerobic capacity). This interpretation aligns with a large‐scale study showing no significant effect of BMI on acute mountain sickness incidence at 4400 m (Song et al., 2014), although the broader literature remains mixed. Collectively, these observations suggest that among healthy, nonobese individuals, BMI is a minor determinant of acute hypoxia tolerance.

Likewise, smoking status did not exacerbate physiological responses to hypoxia. In fact, smokers exhibited slightly attenuated increases in heart rate and blood pressure during exposure. This counterintuitive pattern has been previously reported. For example, Ercan et al. (2021) found that smokers experienced greater acute O_2_ desaturation during hypobaric hypoxia, possibly due to ventilatory stimulation from nicotine or preconditioning from chronic CO exposure. That same study also reported worsened long‐term adaptation in smokers (e.g., elevated pulmonary artery pressures and polycythemia) during prolonged hypoxic exposure (Wu et al., 2012).

In our short 30‐min protocol, the chronically elevated sympathetic tone of smokers may have produced a “ceiling effect,” limiting further rises in HR or BP. Additionally, smokers in our sample were older on average, which complicates interpretation. As shown in our multivariate regression, age—rather than smoking per se—emerged as a more consistent negative predictor of ΔHR, suggesting that chronological aging may underlie blunted cardiac reactivity.

Importantly, SpO_2_ responses did not differ between smokers and nonsmokers, indicating that brief hypobaric hypoxia does not acutely compromise arterial oxygenation in otherwise healthy smokers. All participants experienced similar inspired PO_2_ levels, and the hypoxic ventilatory drive of smokers appeared intact. While it remains possible that chronic smoking‐related impairments (e.g., decreased lung diffusion or pulmonary hypertension) might manifest under longer or repeated exposures, these effects were not observed in our limited‐duration setting.

Finally, it is important to consider selection bias: The smokers in our cohort likely represent a relatively healthy, medically cleared subset of aviators. Therefore, the generalizability of these findings to civilian smokers or individuals with comorbidities remains limited. Further studies are warranted to investigate smoking‐related variability in longer term or operational hypoxia contexts.

Our correlation and regression analyses provide additional insights into the inter‐individual variability in physiological responses. Age was inversely correlated with ΔHR (*r* = −0.27, *p* < 0.05), a finding that is physiologically plausible given that older adults typically exhibit reduced cardiac responsiveness to stress, likely due to diminished *β*‐adrenergic sensitivity and lower maximal heart rate reserve. This aligns with previous high‐altitude studies reporting that older individuals tend to have blunted tachycardia responses but greater or unchanged BP increases, due to age‐related arterial stiffness and higher baseline blood pressure (Mikołajczak et al., 2021).

In our sample, older pilots also had higher resting SBP and DBP, and there was a slight trend toward greater SpO_2_ reduction in those with higher baseline BP, although this did not reach statistical significance. This observation is in line with findings from Mikołajczak et al. (2021), who reported greater hypoxemia under hypoxia in normotensive individuals with elevated BP. However, the magnitude of this effect in our data was modest and did not reach the threshold for clinical relevance.

The strongest correlation in our dataset was observed between baseline SBP and SBP at 5000 m (*r* ≈ 0.74, data not shown), indicating that resting cardiovascular tone largely determines absolute values at altitude, although ΔSBP did not significantly differ across baseline strata. This suggests that individuals with higher baseline SBP maintained proportionally similar BP elevations during hypoxia, consistent with a preserved but shifted hemodynamic set point.

Regarding smoking, we found modest inverse correlations between smoking status and both ΔHR and ΔSBP (*r* = −0.26 for both), suggesting a slightly blunted cardiovascular reactivity in smokers. This may be due to chronic sympathetic activation in habitual smokers, producing a limited reserve for further HR/BP increases under hypoxia. Alternatively, it may reflect an adaptation or tolerance effect, as discussed above.

Importantly, no baseline characteristic—neither age, BMI, BP, nor smoking—significantly predicted the degree of SpO_2_ reduction. This finding supports prior work by Taylor et al. (2025) and Mallet et al. (2021), which demonstrated that oxygen desaturation under acute hypoxia is more strongly influenced by pulmonary and ventilatory factors than by systemic cardiovascular variables. In our uniformly healthy and fit pilot cohort, the drop in SpO_2_ was substantial and consistent across individuals (mean nadir ~77.7%), suggesting a uniform exposure to low alveolar PO_2_ and relatively homogeneous ventilatory responses.

Minor inter‐individual variation in SpO_2_ at altitude likely reflects differences in ventilatory drive, gas exchange efficiency, or pulmonary perfusion, which we did not directly measure. Studies such as that by Fernández‐Rodríguez et al. (2025) similarly concluded that cardiovascular fitness modulates hemodynamic but not desaturation responses, reinforcing this view. Future studies incorporating direct respiratory measures (e.g., respiratory rate, tidal volume, capnography), or exploring genetic markers of hypoxic ventilatory response, may shed light on the residual variability in oxygen desaturation during simulated altitude exposure.

### 4.1 Practical implications

Our findings have several important implications for aviation medicine. Exposure to 5000 m induced marked but transient physiological stress, with mean HR increasing to ~91.9 bpm and blood pressure rising to ~132/82 mmHg—elevations that were statistically significant but physiologically moderate and unlikely to trigger acute cardiovascular events in this healthy pilot population. The pronounced drop in arterial oxygen saturation (mean SpO_2_ ~77.7%) is more concerning from an operational standpoint, as hypoxemia at this level can impair cognition and reaction time if sustained. Indeed, several pilots reported subjective symptoms such as lightheadedness and mild cognitive slowing during plateau exposure, highlighting the practical importance of hypoxia‐awareness training.

Importantly, all physiological responses were fully reversible: within minutes of returning to sea‐level pressure, HR, BP, and SpO_2_ returned to baseline in all participants. This rapid normalization underscores the robust compensatory capacity of healthy aviators and supports the safety and educational value of controlled hypobaric chamber training programs. The absence of adverse events is consistent with prior large‐scale experience in military aviation settings (Minoretti & Emanuele, 2023).

A second practical theme concerns the predictive value of baseline cardiovascular tone. Our analyses showed that higher resting HR strongly predicted smaller HR increases at altitude (*β* = −0.47, *p* < 0.001), while elevated baseline DBP was associated with smaller DBP changes. These findings suggest a ceiling effect or diminished autonomic reserve in individuals with higher resting tone. This phenomenon has been previously observed by Theunissen et al. (2022), Li Volsi et al. (2023), and Beltrán et al. (2020), all of whom reported that baseline sympathetic activity modulates the magnitude of physiological reactivity to hypoxia or other stressors.

Collectively, our data indicate that resting HR and BP could serve as practical screening metrics for predicting the magnitude of acute altitude responses. For example, individuals with high resting HR or BP may benefit from tailored pre‐acclimatization protocols, enhanced in‐flight oxygenation strategies, or stricter hypoxia monitoring thresholds during high‐altitude operations. Integrating such predictive markers into aviation health protocols could enhance both flight safety and individual performance in oxygen‐deprived environments.

### 4.2 Limitations

First, it involved only healthy male pilots, limiting generalizability to other populations, including women and individuals with cardiopulmonary or hematologic conditions. Second, respiratory responses were inferred from SpO_2_ without direct ventilatory measurements (e.g., end‐tidal CO_2_). Third, the 30‐min exposure may not reflect prolonged or repeated high‐altitude stressors.

Fourth, a normoxic control group was not included due to operational and ethical constraints. The hypobaric exposure was part of required health screening, making sham exposures or inclusion of non‐pilot controls impractical. However, the within‐subject design helped mitigate this limitation by allowing each participant to serve as their own control.

Additionally, some confounding may persist, as smokers in the cohort were generally older, despite statistical adjustment. Lastly, minor factors such as chamber noise or movement may have affected BP measurements at altitude, though repeated readings were used to enhance accuracy.

## 5 CONCLUSION

Acute exposure to 5000 m produced significant but fully reversible physiological responses in healthy Vietnamese pilots, including a ~15% increase in heart rate, a moderate rise in blood pressure, and a ~21% drop in oxygen saturation. All parameters returned to baseline within minutes post‐exposure. Traditional risk factors such as higher BMI and smoking status had minimal impact, while older age and elevated baseline cardiovascular tone were associated with attenuated heart rate and BP responses. These findings reinforce the typical pattern of hypoxic adaptation—tachycardia, mild hypertension, and desaturation—while highlighting inter‐individual variation based on baseline physiology.

From an operational perspective, the results affirm the safety and educational value of altitude chamber training in military aviation. By quantifying real‐time cardiopulmonary responses and recovery, this study provides evidence‐based benchmarks to support screening, training, and monitoring strategies in flight environments. Future work should expand to include longer exposures, cognitive effects, and personalized predictive tools for hypoxia tolerance.

## AUTHOR CONTRIBUTIONS

Conceptualization: Phong Nguyen Hong, Nguyen Thanh Xuan, Thuc Luong Cong. Methodology: Phong Nguyen Hong, Nguyen Thanh Xuan, Thuc Luong Cong, Toan Nguyen Duy. Formal Analysis: Phong Nguyen Hong, Nguyen Thanh Xuan, Thao Pham Ngoc, Phuong Nguyen Minh. Investigation & Data Collection: Phong Nguyen Hong, Nguyen Thanh Xuan, Toan Pham Quoc, Toan Nguyen Duy, Luyen Nguyen Van, Thong Nguyen Huy, Thao Pham Ngoc. Writing – Original Draft: Phong Nguyen Hong, Nguyen Thanh Xuan, Toan Pham Quoc, Luyen Nguyen Van, Thong Nguyen Huy. Writing – Review & Editing: Phong Nguyen Hong, Nguyen Thanh Xuan, Thuc Luong Cong, Tuan Tran Ngoc, Phuong Nguyen Minh, Toan Pham Quoc, Toan Nguyen Duy, Thong Nguyen Huy, Thao Pham Ngoc, Luyen Nguyen Van. Supervision: Phuong Nguyen Minh, Tuan Tran Ngoc. All authors have read and agreed to the published version of the manuscript.

## FUNDING INFORMATION

This research did not receive any specific grant from funding agencies in the public, commercial, or not‐for‐profit sectors.

## CONFLICT OF INTEREST STATEMENT

The authors declare that they have no conflicts of interest relevant to this study.

## ETHICS STATEMENT

The study protocol was approved by the Institutional Review Board (IRB) of the Vietnam Military Medical University (Approval No. 4050/QĐ‐HVQY, dated 20/09/2024) and was endorsed by the Vietnam Air Force Institute of Aviation Medicine. All participants provided written informed consent before participating in the study.

## Data Availability

The data supporting the findings of this study are available from the corresponding author on reasonable request. Due to privacy and ethical restrictions, individual participant data are not publicly shared.
